# An overview of the cutaneous porphyrias

**DOI:** 10.12688/f1000research.10101.1

**Published:** 2017-10-30

**Authors:** Robert Dawe

**Affiliations:** 1 Scottish Cutaneous Porphyria Service, Scottish Photodiagnostic Unit, Department of Dermatology, Ninewells Hospital and Medical School, Dundee, DD1 9SY, UK

**Keywords:** porphyrias, enzyme defects, hereditary, symptoms

## Abstract

This is an overview of the cutaneous porphyrias. It is a narrative review based on the published literature and my personal experience; it is not based on a formal systematic search of the literature. The cutaneous porphyrias are a diverse group of conditions due to inherited or acquired enzyme defects in the porphyrin–haem biosynthetic pathway. All the cutaneous porphyrias can have (either as a consequence of the porphyria or as part of the cause of the porphyria) involvement of other organs as well as the skin. The single commonest cutaneous porphyria in most parts of the world is acquired porphyria cutanea tarda, which is usually due to chronic liver disease and liver iron overload. The next most common cutaneous porphyria, erythropoietic protoporphyria, is an inherited disorder in which the accumulation of bile-excreted protoporphyrin can cause gallstones and, rarely, liver disease. Some of the porphyrias that cause blistering (usually bullae) and fragility (clinically and histologically identical to porphyria cutanea tarda) can also be associated with acute neurovisceral porphyria attacks, particularly variegate porphyria and hereditary coproporphyria. Management of porphyria cutanea tarda mainly consists of visible-light photoprotection measures while awaiting the effects of treating the underlying liver disease (if possible) and treatments to reduce serum iron and porphyrin levels. In erythropoietic protoporphyria, the underlying cause can be resolved only with a bone marrow transplant (which is rarely justifiable in this condition), so management consists particularly of visible-light photoprotection and, in some countries, narrowband ultraviolet B phototherapy. Afamelanotide is a promising and newly available treatment for erythropoietic protoporphyria and has been approved in Europe since 2014.

## An overview of the cutaneous porphyrias

The porphyrias affecting the skin can be divided into (1) those that present mainly with visible light–exposed site bullae and fragility (most commonly porphyria cutanea tarda [PCT]), (2) those, particularly erythropoietic protoporphyria (EPP), that present with neuropathic-type pain, oedema, erythema, and lesions on exposed sites (acute phototoxic porphyria), and (3) the extremely rare, severe, and mutilating porphyrias such as congenital erythropoietic porphyria (CEP; Günther’s disease). There are various classification schemes, but if the porphyrias are considered from the point of view of the clinical cutaneous features, this is now one of many porphyria classification schemes ^1, 2^, along with considerations of where in the body overproduction of porphyrin intermediates occurs (hepatic or erythropoietic) and whether it is mainly a neurovisceral attack porphyria or a cutaneous porphyria or where (which country) it was first described in detail (such as South African porphyria and Swedish porphyria).

For those not familiar with the cutaneous porphyrias, important general points are that (1) they are a diverse group of conditions, all showing skin diseases strictly limited to sun-exposed skin areas with a predilection for the backs of the hands and the face, and are due to a problem in the haem biosynthetic pathway—so certain types of cutaneous porphyria rather than ‘cutaneous porphyria’ should be part of a differential diagnosis list—(2) it is visible light (not ultraviolet) that is relevant, and (3) all the cutaneous porphyrias can also be associated with complicating diseases in organs other than the skin.

**Figure 1.**
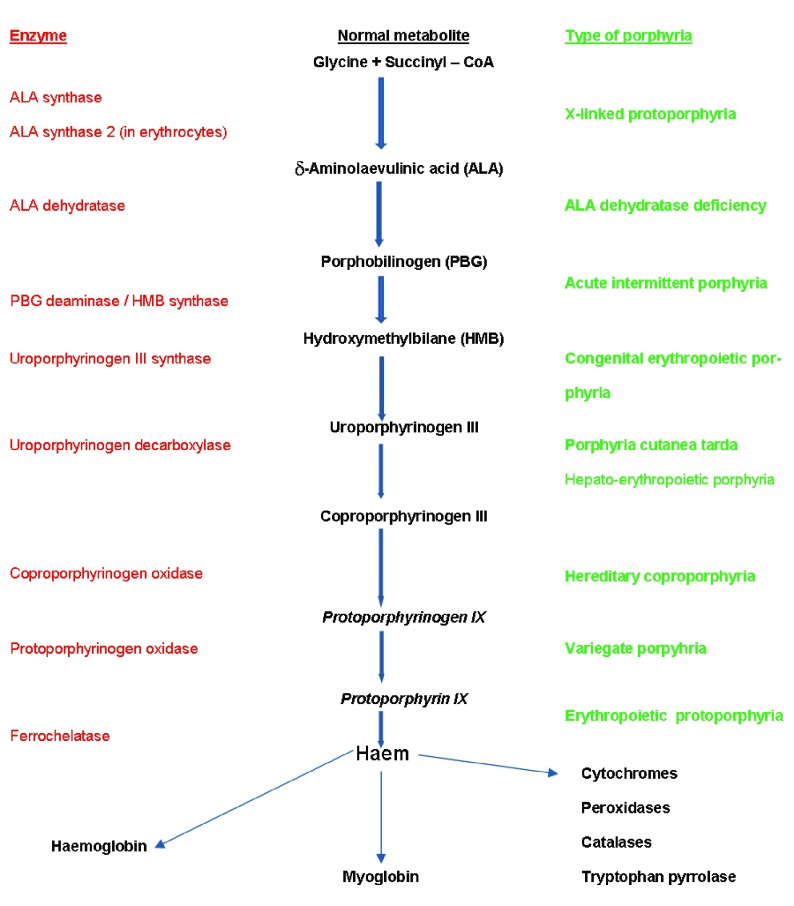
Simplified porphyria–haem biosynthetic pathway illustrating the enzyme defects (decreased activity for all except aminolevulinic acid (ALA) synthase 2 with which increased activity causes X-linked protoporphyria) involved in the main porphyrias.

The porphyrias are a group of conditions caused by inherited or acquired enzyme defects in the haem biosynthesis metabolic pathway ( Figure 1). All the porphyrias except acute intermittent porphyria and the exceptionally rare aminolevulinic acid (ALA) dehydratase deficiency porphyria (Doss porphyria) can affect the skin.

Two of the blistering (usually bullae, large blisters) and fragility (variegate porphyria [VP] and hereditary coproporphyria [HC]) porphyrias can also cause acute porphyria attacks. Acquired PCT is the one porphyria that is not primarily inherited but is a consequence of chronic liver inflammation and liver iron overload. EPP can affect the biliary system and rarely the liver and is frequently associated with a mild anaemia.

The three commonest cutaneous porphyrias in the UK are PCT, which affects about 1 in 13,000 people in Scotland; EPP, which affects about 1 in 43,000 people in Scotland; and VP, which affects about 1 in 240,000 people in Scotland ^3^. HC has skin manifestations identical to those of PCT and VP. The rare CEP and hepatoerythropoietic porphyria (homozygous inherited PCT) are usually severe but exceptionally rare (affecting less than one in a million ^3^) diseases. Certain chemicals can induce a porphyria, such as hexachlorobenzene, a pesticide that caused an epidemic of a porphyria, with cutaneous features akin to PCT, in Turkey ^4^. The term pseudoporphyria is usually used when porphyria is excluded as the cause of a PCT-like presentation, but the term is also used to encompass some features more similar to EPP, such as in children taking naproxen ^5^, and is usually a drug-induced adverse effect (commonly caused by a non-steroidal anti-inflammatory drug and often naproxen), although it has also been reported with high cumulative exposure to sunbed radiation in the absence of a recognised drug trigger. In kidney failure, a PCT-like presentation may not be due to an abnormality in porphyrin–haem metabolism or to a drug but to raised porphyrins due to failure to excrete them.

Diagnosis of the porphyrias is as with other conditions: it starts with history and examination. If a cutaneous porphyria like PCT is in the differential, a porphyrin plasma scan (plasma spectrofluorimetry) and quantitative urine porphyrins are appropriate initial tests ^6^. If these are positive, then fluorescent high-performance liquid chromatography on urine and stool porphyrins are appropriate in order to differentiate among PCT, VP, and HC. If acquired PCT is strongly suspected, it is appropriate to start investigating for underlying causes, including iron accumulation, chronic hepatitis B or C, or HIV, even while still investigating to determine whether the patient does have PCT. If EPP is considered, then it is appropriate to request a porphyrin plasma scan and quantitative erythrocyte protoporphyrin.

## Acute phototoxic symptoms

### Erythropoietic protoporphyria

EPP, which is due to reduced ferrochelatase activity ( Figure 1), is the second most common cutaneous porphyria, affecting about 2.3 per 100,000 population in the Dundee area ^3^. This estimate is higher than a survey of diagnosed UK cases suggested (0.86 per 100,000); similar discrepancies were seen in prevalence estimates for the whole of Denmark compared with a region in Denmark, including a centre with a particular interest in this condition ^7^.

EPP is the most frequent porphyria to present in childhood. Its clinical features are very different from those of the cutaneous porphyrias that present mainly with blistering and fragility. Rarely, a form of EPP can occur as an acquired problem, usually associated with myelodysplastic conditions such as sideroblastic anaemia ^8^. Recently, a condition that used to be grouped together with EPP, X-linked protoporphyria, has been distinguished (see below). Within the differential, there are sometimes certain patterns of drug-induced phototoxicity and atypical polymorphic light eruption. Also in the differential, when EPP induces urticaria, it is usually idiopathic solar urticaria. In fact, EPP was originally classified as type VI solar urticaria ^9, 10^.

The main feature is pain, of a burning or prickling quality, in skin exposed to as little as a few minutes of sunlight (which might be cloud or window glass–transmitted, as it is visible radiation that is predominantly responsible for the exposed site skin symptoms in this, as in the other, porphyrias). Typically, the pain is severe, and those affected withdraw from further exposure if possible and often try to obtain some relief by various manoeuvres, often running cold water or applying cold packs on skin or sometimes using a cream containing menthol or similar over-the-counter counter-irritant topical preparations such as ‘Tiger Balm’ (Haw Par Corporation, Singapore).

Only if further exposure is unavoidable will swelling appear as a result of endothelial damage, leading to increased dermal tissue fluid. Sometimes redness appears and purpura can occur if there is sufficient damage to the endothelium that red blood cells as well as dermal tissue fluids leak into the dermis. With repeated exposures of this kind, there can be a typical linear (but rather chicken pox-like) scarring and a waxy thickening of skin, especially over the dorsal nose and knuckles. Other features, such as rhagades, can develop ^11^. Palmar keratoderma is especially associated with classic autosomal recessive inheritance ^12^.

This condition typically presents first in childhood, with the parents noticing that a baby cries when exposed to sunlight. Because of the frequent absence of physical signs, there is often a very prolonged delay in diagnosis; in a survey of most of the UK EPP patients, a delay of a median of 12 years before diagnosis was reported ^11^. In countries with compulsory national service, EPP is one of those conditions that can initially be mistaken for malingering.

Inheritance is now recognised in about 9 out of 10 people with EPP to be ‘autosomal co-dominant’ (a form of autosomal recessive) where a major sporadic mutation in the gene encoding ferrochelatase is inherited from one parent and a polymorphism is inherited from the other parent. A common polymorphism that leads to slightly reduced ferrochelatase activity is frequent and found in almost 11% of a western European population and at higher frequency in a Japanese population, mainly accounting for the worldwide distribution of the prevalence of EPP ^13, 14^.

Apart from the skin features, gallstones are common and can become troublesome. A mild anaemia, often of a hypochromic microcytic pattern, is frequent ^15, 16^. The main relevance of this is that if this anaemia is treated with iron, this can be counterproductive. Low iron stores appear to be protective in erythropoietic porphyrias ^17^.

EPP liver failure is not common: two cases were found in a study of almost 400 UK patients ^11^, and I know of one patient in Scotland who died of protoporphyria liver failure within the past 25 years. This is an important complication to be aware of, however, as it can start seemingly rather innocuously with only a moderate rise in transaminases that in other situations (such as suspected alcoholic liver disease) would not generally cause much concern but in EPP should prompt early action. The review by Anstey and Hift in the *Postgraduate Medical Journal* ^18^ and *Gut* ^19^ is helpful.

The diagnosis can be made on the basis of symptoms, an abnormal plasma scan (peak emission at 630 to 635 nm), and elevated red blood cell (erythrocyte) protoporphyrin. On a whole blood scan, most of the excess protoporphyrin is unchelated. At diagnosis, it is usual to check full blood count and liver function tests (LFTs). It is advisable to monitor with LFTs, although there is no consensus on what tests should be done or how frequently ^18, 19^. I usually recommend 6-monthly LFTs and erythrocyte protoporphyrins in an attempt to pick up early any evidence of liver failure developing or marked increase in porphyrin that might be associated with an increased risk of subsequent liver failure.

Management involves telling the patient the diagnosis, genetic counselling as appropriate to the individual’s situation, and advice on environmental, behavioural, clothing, and topical sunscreen photoprotection. It is important to explain to patients that it is visible light that causes the skin symptoms and to tailor advice to reducing visible-light exposure. This may be obvious to patients, but in Scotland many initially think that it must be ultraviolet—because almost everyone is familiar with normal sunburn, caused mainly by ultraviolet B (UVB)—that is relevant, and a lot is more understandable to them once they understand that this is a visible-light problem. ‘Daylight’, at least in Scotland, is a term that can help people understand the distinction from the ultraviolet in sunlight that causes sunburn. For example, most commercially available sunscreens are minimally effective in visible-wavelength problems, including the cutaneous porphyrias. In fact, most commercial sunscreens that contain reflective particles such as titanium dioxide deliberately use small particle sizes to improve their efficacy in ultraviolet protection and reduce their reflection of visible light (as such reflection of wavelengths that the human eye can see unavoidably makes a sunscreen messier). However, a large-particle-size titanium dioxide sunscreen (such as that made by the UK National Health Service orphan drug manufacturer Tayside Pharmaceuticals) can be of benefit for visible-light photoprotection if used along with other measures ^20^.

All medical interventions for the prevention of the cutaneous phototoxicity of EPP, with the exception of afamelanotide (see below), have been poorly studied ^21^. Beta-carotene has often been tried, but its true efficacy is uncertain, as complete blinding in studies is impossible because of the skin colour changes produced at doses that might work ^22– 25^.

The study by Corbett *et al*. was a randomised crossover study with blinding maintained when possible. I performed some analyses myself on their published data, as confidence intervals were not presented and *P* values were just given as <0.05 or not without more detail ^24^. Fourteen people with EPP were recruited and 11 completed the crossover study (random order of allocation to placebo or beta-carotene): the authors reported no detectable difference in one main outcome measure (‘hours out of doors’) and reported being unsure of the accuracy of patient recording of the other main outcome measure recorded (on diary cards), ‘hours outdoors in bright sunlight’. Patients reported a mean of 23.1 ‘hours outdoors in bright sunlight’ over 5 weeks on beta-carotene compared with a mean of 15.7 hours over 5 weeks on placebo; that is, on beta-carotene, a mean of 7.4 (95% confidence interval 1.1 to 13.7) more hours were recorded ‘outdoors in bright sunlight’ ( *P* = 0.026). The authors considered that although this difference was statistically significant (unlikely due to chance), it was likely to be ‘clinically insignificant’. Other antioxidants have been tried, but controlled crossover studies (such as that by Bijlmer-Iest *et al*. ^26^) did not detect benefit with N-acetylcysteine, and although another small study showed possible benefit with vitamin C, this drug has not continued to be used routinely in the centre that performed the study ^27^. H1 antihistamines can reduce the weal-flare response in solar urticaria occurring as a manifestation of EPP ^28^.

Narrowband UVB phototherapy can provide useful protection presumed in part to be through inducing epidermal thickening and also *possibly* through increased pigmentation (tanning) ^29– 31^, although it is unlikely that this is a major way in which it works. A personal clinical observation from working in an area where many are of sun-reactive skin phototype I (and so do not tan with sunlight or UVB phototherapy) is that there does not seem to be any connection between tanning and a good response to treatment. Narrowband UVB lamps (TL-01 lamps, Philips, Eindhoven, The Netherlands) emit some visible light along with the UVB, but in practice there is proportionately so little visible light that this treatment does not cause EPP phototoxicity. In a review of 80 courses of UVB given to 12 patients (of whom seven reported a ‘good benefit’), only two experienced EPP symptoms due to daylight exposure, not due to treatment, while attending for narrowband UVB phototherapy ^30^. In that case series, two had episodes of well-demarcated erythema after UVB; this was sunburn-like UVB erythema, not EPP phototoxicity. Anecdotally, when EPP patients experience normal mild sunburn during a course of UVB, they are often pleased, having never experienced normal sunburn before (because the EPP phototoxicity has previously prevented sufficient sunlight exposure to allow doses of normally erythemogenic wavelengths to cause this). Possibly, part of the way narrowband UVB works is through the same main mechanism that is thought to explain its efficacy in chronic urticaria, that of mast cell stabilisation ^32^. There is some *in vitro* evidence that after porphyrin absorbs visible light, later degranulation of mast cells is an important part of the process ^33, 34^. There are many other effects, beyond simple physical ‘hardening’, of UVB which could contribute to its effectiveness.

Afamelanotide, an alpha-melanocyte stimulating hormone (α-MSH) analogue, improves skin photosensitivity in EPP ^35^. It increases eumelanin production, which may be of benefit partially through direct photoprotection effects and also through the effects of eumelanin as a free radical scavenger. Moreover, α-MSH modulates skin inflammation and increases antioxidant enzymes. It causes tanning, making blinded study difficult. Its very long-term safety is not yet known, although a study with up to 8 years of experience has been published ^36^. In a multicentre randomised European study, those on afamelanotide reported a median of 6 (range of 0 to 193) hours daily outside in direct sunlight over the 9 months of the study without pain compared with 0.8 (range of 0 to 35) hours ( *P* = 0.005) amongst those allocated placebo, and in a USA study, those allocated afamelanotide reported a median of 69.4 (range of 0 to 651) hours out in direct sunlight without pain over 6 months of study compared with a median of 40.8 (range of 0 to 224) hours ( *P* = 0.04) amongst those allocated placebo ^37^. There are reports of 93% of treated EPP patients having a marked response, and those on it tend to stay on it ^36^, so this may turn out to be a highly useful treatment. Also, study is difficult, particularly because people with EPP become frightened of sunlight exposure, so in the randomised studies (Europe and the USA) patients may still have gone out less than they would have been able to for fear of painful reactions, so this study endpoint might not have fully reflected the benefits of afamelanotide. Conversely, perhaps many of those who received afamelanotide guessed they were on active treatment because of tanning and so went out longer (so possibly gaining benefit through greater exposure to sunlight ^38^).

This drug is now licensed (and has marketing authorisation for the prevention of phototoxicity in adult EPP patients) in Europe. It is already in use in some countries and in others is currently being considered by bodies such as the Scottish Medicines Consortium. Ideally, I think, there should now be a study directly comparing it with another intervention such as narrowband UVB (perhaps given as home phototherapy). Langendonk *et al*. have shown that large multicentre studies in such a rare condition can be done ^37^. Others think that first a study on the effectiveness and safety of UVB using a double-blind design should be performed.

Cimetidine has been tried and reported to be helpful for some ^39– 41^, although these cases were not clearly reported ^42^. *If* it really works, it is more plausible that it is working as an H2 antihistamine in the skin, as in solar urticaria ^43^, rather than working through inhibiting ALA synthase. It is hepatic ALA synthase 1, not erythroid ALA synthase 2, that it inhibits.

Various measures to increase protoporphyrin elimination in bile—such as colestyramine (cholestyramine) and activated charcoal—have been tried for incipient liver failure ^17, 18, 44, 45^. Colestyramine does not appear to have an effect on red cell porphyrins in EPP ^46^. Liver transplantation has saved the lives of patients with liver failure ^47^, although EPP symptoms continue after such a transplant and, as the recurrence rate of liver disease is high, bone marrow transplantation has been conducted at the same time as liver transplantation to prevent future failure of the transplant due to protoporphyria liver disease ^47, 48^. If a patient with EPP does need surgery, particularly for liver transplantation that is being conducted because of EPP liver failure (when porphyrin levels are typically especially high), operating theatre lamps should be shielded to particularly reduce violet and blue wavelengths of visible light ^49^.

### X-linked protoporphyria

This has recently been described as a distinct disease ^50^. In the past, most patients with this condition were probably considered to have classic EPP. In this condition, in contrast to EPP, there is not a reduced activity of the ferrochelatase enzyme but instead an increased activity of the ALA synthase 2 enzyme, which is expressed only in the red blood cell line ( Figure 1). So, in this condition, problems caused by increased protoporphyrin are caused by total increased protoporphyrin production rather than insufficient enzyme activity to chelate iron into protoporphyrin. This is relevant regarding diagnosis. In classic EPP, there is a marked increase in free protoporphyrin, whereas in X-linked EPP there is also an increase in zinc chelated protoporphyrin; this is because the ferrochelatase enzyme is of normal activity and able to chelate some of the excess protoporphyrin with divalent metals such as zinc. Possibly in this condition, unlike in classic EPP, iron supplementation may turn out to be a useful treatment ^51^.

## Bullae and skin fragility

### Porphyria cutanea tarda

This is the commonest of the cutaneous porphyrias in Europe and probably worldwide. The prevalence in Dundee is 1 in 13,000 ^3^. PCT is important not only in its own right but also because sporadic PCT (sometimes called type 1 PCT) is a marker for liver disease and an increased risk of hepatocellular carcinoma. PCT is caused by a reduction in the activity of uroporphyrinogen decarboxylase ( Figure 1), and different inherited patterns of this deficiency give rise to the rare types (2 and 3). Most cases of PCT are type 1, which is also known as ‘sporadic’ or ‘acquired’. In these patients, liver disease and iron overload are necessary to reduce this enzyme activity level sufficiently for PCT to develop.

When chronic hepatitis induces PCT, liver iron overload is usually present, and heterozygosity for a haemochromatosis gene contributes to PCT development in many affected individuals ^52, 53^. In Europe, the most common causes of PCT are excessive alcohol consumption, chronic hepatitis C infection, other liver-related chronic viral infections (including HIV), autoimmune chronic hepatitis (for example in systemic lupus erythematous), and haemochromatosis. Medications are not triggers in contrast to the acute porphyrias (see below), except for oestrogens that may play a part in inducing PCT in some people ^54^, and treatment with iron will exacerbate PCT.

Sporadic PCT usually first develops over the age of 40 and seems to be more common in men than in women. The most frequent presentation is with blisters, skin fragility, and delayed healing of wounds on the maximally photo-exposed sites of dorsal hands. Upon healing, the subepidermal blisters of PCT typically result in milia; classically, these are seen on the maximally photo-exposed sites of the dorsal hands. In most PCT patients, this means most frequent localisation over dorsal thenar eminences, proximal index and middle fingers, and thumbs. Different distributions can be seen dependent upon the patient’s occupation; for example, I recently saw a taxi driver with more involvement of distal dorsal fingers and with finger nail changes, including onycholysis. Hypertrichosis also frequently appears; it often first manifests on the temples but can appear in areas that are not photo-exposed. Another frequent feature is pigmentation, particularly of areas that are photo-exposed, and is sometimes the only presenting sign. Solar urticaria is rarely seen on presentation ^55, 56^, although urticarial responses can be found on monochromator phototesting ^57^.

Scleroderma-like changes are infrequent in PCT but have been thoroughly reported ^58^. Many PCT sufferers are not initially aware of the involvement of sunlight. This is because (1) it is visible (not ultraviolet) wavelength radiation that leads to the changes in the skin, so seasonal variation is not always apparent, and (2) it is mainly chronic, and not discrete episodes of, sunlight exposure that result in symptoms.

When one is evaluating a patient exhibiting typical features, the primary differential diagnoses to consider are VP, HC, and drug-induced pseudoporphyria. A peak at around 620 nm on plasma spectrofluorimetry is present in both PCT and HC. The diagnosis of PCT can be corroborated by quantification of urine and stool porphyrins. PCT patients excrete increased amounts of isocoproporphyrin in faeces, whereas both VP and HC patients show a strongly increased coproporphyrin III isomer. Urinary porphyrin concentrations can be used to monitor the response to therapy. In an unusually young patient (younger than 30 years old) presenting with PCT, the mainly inherited PCT types should be taken into account. In most cases, sporadic PCT is the diagnosis, and subsequently the underlying liver insults and whether or not there is clear evidence of iron overload must be determined. All patients are investigated for transaminases, hepatitis B and C serology, ferritin, and alfa-fetoprotein (because of the risk of hepatocellular carcinoma).

A liver ultrasound is often done (particularly to look for hepatocellular carcinoma) but liver biopsy is rarely indicated. In areas where there is a high prevalence of HIV, patients should always undergo HIV serologic testing. In areas where there is a low prevalence, the occurrence of HIV testing is expected to be low, but investigation must still take place if there are other pointers to this diagnosis (such as mouth ulcers, chronic diarrhoea, weight loss, or behavioural risk factors) or, regardless of the presence or absence of behavioural/lifestyle risk factors, if the PCT presentation is unusual (for example, in a young woman who drinks little alcohol) and initial investigations have been unrewarding.

Management involves eliminating the causes of underlying liver disease and iron overload when possible. Patients should be advised to avoid alcohol (irrespective of whether or not it is thought to be the cause of the primary liver insult), medications containing iron must be stopped, and oestrogens should be stopped or changed to low systemic dose transdermal preparations. When possible, viral hepatitis should be treated, and several PCT patients I have been following up over the past two decades have been cured by some of the newer anti–hepatitis C drugs. The likelihood of hepatitis C treatment being effective is possibly increased if iron overload is addressed first ^59^. Iron overload is usually treated by venesection. Iron chelation therapy (with desferrioxamine or one of the newer oral iron chelators) is an alternative approach that can be useful when iron overload is associated with anaemia (such as in a haemodialysis patient with PCT ^60^).

Another approach, which can be combined with venesection, is to treat with low-dose chloroquine or hydroxychloroquine. These drugs are thought to work by increasing porphyrin excretion. Chloroquine rather than hydroxychloroquine is usually used, as there is more experience in using this in PCT and the doses used (such as 250 mg chloroquine phosphate weekly) are low enough that ocular toxicity is not an important concern ^61^. Owing to the risks of severe hepatotoxicity, higher doses should not be used. While patients await the effects of these treatments, adopting sunlight avoidance measures is important. Visible-light exposure can be lowered by behavioural avoidance, wearing suitable clothing (for example, dark-coloured driving gloves), and applying a large-particle-size titanium dioxide sunblock. Also, protecting the hands from trauma during manual work and leisure activities (such as gardening) by wearing gloves is beneficial. For male patients, the use of an emulsifying ointment as an alternative to shaving foam, applied for 10 minutes before shaving, can help reduce facial shaving trauma.

### Acute porphyrias with skin manifestations (variegate and hereditary coproporphyria)

Cutaneous features of VP and HC can be identical to those of PCT. Features of an acute attack include gastrointestinal symptoms (abdominal pain, nausea, vomiting, constipation, and diarrhoea), neurological symptoms (motor and sensory peripheral neuropathies, muscle pain, ascending paralysis, anxiety, psychosis, coma, seizures, and respiratory paralysis requiring ventilation), cardiovascular symptoms (tachycardia and hypertension), and renal failure ^62^.

The Norway-based NAPOS (http://www.drugs-porphyria.org) porphyria website can provide useful guidance when prescribing drugs to people with acute attack porphyrias and when considering possible triggers for an acute attack. Those affected who have had multiple attacks will often have learnt which drugs alleviate their symptoms and are safe for them. Such patients presenting to a hospital where they are not known, and requesting a high dose of a particular opiate and an anti-emetic such as chlorpromazine, may initially be suspected of being drug addicts.

Screening of family members of VP- and HC-index patients should be undertaken. In VP, a negative plasma fluorescence test is still associated with a 1-in-8 risk for a first-degree relative over 15 years of age; a second-degree relative has only a 1-in-22 risk (close to the certainty achieved by measuring protoporphyrin oxidase enzyme activity) ^63^. Previously, genetic testing was difficult, except in those from the Western Cape in South Africa, where one particular mutation accounts for the majority of cases ^64^, but genetic testing is now more generally useful ^65^ and is the standard approach for screening of family members.

Management of the cutaneous aspects of these porphyrias involves the same advice on reducing visible-light exposure as for the other porphyrias. Acute attacks are managed as for acute intermittent porphyria. That is, efforts must be taken to avoid inducing attacks (such as by prescribing porphyrinogenic drugs) and to prevent attacks being set off by known triggers (such as dieting). Oral tin-protoporphyrin may (by negative feedback reducing ALA synthase activity) help prevent acute attacks ^66, 67^ but is itself a toxic photosensitiser that can cause symptoms akin to those of EPP and so is not used now. Through a direct inhibition of the haem biosynthetic pathway (particularly reduced ALA synthase activity), carbohydrate loading can also be useful in less severe attacks or as an attack preventative measure. Intravenous haem arginate (a haem analogue) aborts acute attacks and should be considered for acute attacks, including for those who have had previous life-threatening attacks. In some countries, such as in England and Wales (Scotland will be joining), there is an acute porphyria service which can help in advising whether haem arginate should be used and getting it supplied if recommended.

## Severe scarring porphyrias

### Congenital erythropoietic porphyria (Günther’s disease)

CEP is very rare, affecting less than 1 in a million in Scotland ^3, 68^. Severe photosensitivity occurs with phototoxicity, blistering, chronic ulcers, and delayed healing and mutilation of light-exposed parts. Hypersplenism, haemolytic anaemia with thrombocytopaenia, may occur. Erythrodontia, brown teeth that fluoresce under Wood’s lamp (a violet lamp with peak irradiance at about 410 nm) illumination, is another sign. Milder variants with onset in adult life can occur and have presented with and without thrombocytopaenia ^69, 70^. Management of severely affected individuals means strict attempts to avoid visible solar radiation reaching the skin and eyes. Bone marrow transplantation is used for the most severe cases. Katugampola *et al*. have suggested a plan for the multidisciplinary management of people with CEP and criteria to consider when determining whether bone marrow transplantation is appropriate ^71^.

### Homozygous and mixed porphyrias

These tend to present early, and features include scarring (frequently causing scarring alopecia and syndactyly) in many patients. Hepatoerythropoietic porphyria (homozygous inherited PCT) can present with the clinical features of CEP but the biochemistry of PCT. Homozygous VP is now well characterised ^72^, presenting in childhood, with short stature, and photosensitivity with features suggesting very severe PCT, including scarring and clinodactyly. Homozygous VP has not been associated with acute neurovisceral attacks.
